# Prospective Trial of CPAP in Community-Dwelling Adults with Down Syndrome and Obstructive Sleep Apnea Syndrome

**DOI:** 10.3390/brainsci10110844

**Published:** 2020-11-12

**Authors:** Elizabeth A Hill, Donna M Fairley, Linda J Williams, Goffredina Spanò, Sally-Ann Cooper, Renata L Riha

**Affiliations:** 1 Sleep Research Unit, Centre for Clinical Brain Sciences, University of Edinburgh, 51 Little France Crescent, Edinburgh EH16 4SA, UK; lizzie.hill@ndcn.ox.ac.uk (E.A.H.); donnamfairley@aol.com (D.M.F.); 2 Centre for Population Health Sciences, Usher Institute, Old Medical School, University of Edinburgh, Teviot Place, Edinburgh EH8 9AG, UK; linda.williams@ed.ac.uk; 3 Down Syndrome Research Group, Department of Psychology, University of Arizona, Tucson, AZ 85721, USA; gspano@email.arizona.edu; 4 Mental Health and Wellbeing Research Group, Institute of Health and Wellbeing, University of Glasgow, Glasgow G12 0XH, UK; sally-ann.cooper@glasgow.ac.uk

**Keywords:** obstructive sleep apnoea syndrome, developmental behaviour checklist-adults, down syndrome, continuous positive airway pressure, cognition, cognitive testing in down syndrome, adults, pilot randomised controlled trial

## Abstract

Adults with Down syndrome (DS) are predisposed to obstructive sleep apnoea (OSA), but the effectiveness and acceptability of continuous positive airway pressure treatment (CPAP) in this group has rarely been formally assessed. This study was designed as a pilot randomised, parallel controlled trial for one month, continuing as an uncontrolled cohort study whereby the control group also received the intervention. Symptomatic, community-dwelling DS individuals exhibiting ≥10 apnoeas/hypopneas per hour in bed on a Type 3 home sleep study were invited to participate in this study, with follow-up at 1, 3, 6, and 12 months from baseline. Measurements of sleepiness, behaviour, cognitive function and general health were undertaken; the primary outcome was a change in the pictorial Epworth Sleepiness Scale (pESS) score. Twenty-eight participants (19 male) were enrolled: age 28 ± 9 year; body mass index 31.5 ± 7.9 kg/m^2^; 39.6 ± 32.2 apnoeas/hypopneas per hour in bed; pESS 11 ± 6/24. The pilot randomised controlled trial at one month demonstrated no change between the groups. At 12 months, participant (*p* = 0.001) pESS and Disruptive (*p* < 0.0001), Anxiety/Antisocial (*p* = 0.024), and Depressive (*p* = 0.008) behaviour scores were reduced compared to baseline. Improvement was noted in verbal (*p* = 0.001) and nonverbal intelligence scores (*p* = 0.011). General health scores also improved (*p* = 0.02). At the end of the trial, 19 participants continued on treatment. Use of CPAP in adults with DS and OSA led to a number of significant, sustained improvements in sleepiness and behavioural/emotional outcomes at 12 months.

## 1. Introduction

Down syndrome, present in one in 1000 live births worldwide [1], is the commonest form of intellectual disability. Current estimates suggest that >47,000 people in the UK and >250,000 people in the USA have Down syndrome [2,3]. 

Sleep-disordered breathing, characterised by repetitive pauses in breathing during sleep, affects 24% of the general adult population [4]. Obstructive sleep apnoea syndrome (OSAS) is diagnosed when nocturnal apnoea results in significant diurnal symptoms, including excessive daytime somnolence, impaired cognitive function, reduced quality of life, and behavioural and emotional disturbances [5]. The adult prevalence of OSAS in the general population is 5–7% [5], rising to 35–37% in adults with Down syndrome [6]. OSAS is considered an independent risk factor for cardiovascular morbidity and mortality, including hypertension, myocardial infarction and stroke [5,7].

The Down syndrome phenotype includes a flattened face, short neck, generalised hypotonia, loose ligaments, and a tendency toward weight gain—all risk factors for OSAS. Down syndrome causes cognitive impairment per se, so additional consequences of OSAS may conceivably be more profound due to lack of existing cognitive reserve [8]. 

Continuous positive airway pressure (CPAP) therapy is the gold-standard treatment for moderate-severe OSAS in adults, and is effective in ameliorating OSAS consequences, including excessive daytime, cognitive dysfunction and metabolic and cardiovascular outcomes in the general population although the data are not always consistent [9,10,11,12]. CPAP use has been shown to reduce the risk of all-cause mortality to a similar level as the general, non-OSAS, population [13]. However, despite the potential benefits, diagnosis and treatment of OSAS in adults with Down syndrome is not commonplace, and, to date, no objective studies of CPAP effectiveness in the Down syndrome population have been published. This study aimed to assess the effectiveness and acceptability of CPAP in adults with Down syndrome and OSAS living in the UK, with reference to subjective sleepiness, behavioural and emotional function, cognitive function, and general health.

## 2. Methods 

This study was designed as a one-month randomised, controlled pilot trial of CPAP (Trial registration: ISRCTN55685305), with a 12-month cohort study follow-up, incorporating repeated measures. Enrolment and testing of participants took place out with medical institutions in all four nations of the United Kingdom. 

Apart from a clinical diagnosis of Down syndrome, inclusion criteria were age ≥16 years (considered to be legal adulthood in Scotland) [14], and ≥18 years of age in England, Northern Ireland, and Wales.

The Scotland A Research Ethics Committee approved the study, registered as ISRCTN55685305. 

Recruitment was undertaken in the context of a larger study conducted by us on the objective and subjective prevalence of OSAHS in adults with DS [15]. Briefly, questionnaires and pre-paid reply envelopes were sent to 5266 UK-based adults with Down syndrome and their caregivers between 14.02.2011 and 10.01.2014. Potential study participants were identified by local and national organisations supporting people with Down syndrome (see Acknowledgements). The questionnaire comprised a section for completion by the individual with Down syndrome and a section for completion by a relative/caregiver. Anthropometric, comorbidity, medication, demographic, and sleep disturbance data (including frequency per week of snoring, witnessed apnoeas, nocturnal choking episodes, frequent awakenings, unrefreshing sleep and daytime sleepiness) were collected. The pictorial version of the Epworth Sleepiness Scale (pESS), [16] designed to enhance understanding and accessibility in a broader adult population was also administered.

Based on the presence of symptoms commensurate with possible OSAS, participants were then invited to undertake a home sleep study which was conducted using the Embletta^®^ Gold™ (Embla Systems LLC, Amsterdam, the Netherlands) cardio-respiratory polygraphy device. This is a Type3 device [17,18], with capacity to record multiple channels of physiological data. Home sleep apnoea testing using Type3 polygraphy is recommended in national guidelines [18] and is routinely used in clinical practice across the UK. Channels used in broad accordance with the AASM guidelines for portable monitoring [18] were nasal airflow and snoring recorded via nasal pressure cannula, respiratory effort recorded via thoracic and abdominal respiratory inductance plethysmography bands, SpO_2_ recorded via pulse oximetry and body position recorded via an inbuilt position sensor. All studies were manually validated and scored by one of two experienced Registered Polysomnographic Technologists using standard software (Embla^®^ RemLogic™ Embla Systems LLC, Amsterdam, The Netherlands) in broad accordance with current international guidelines [17]. To ensure consistency of scoring, inter- and intra-rater reliability scoring was conducted in randomly selected subsets of 10% of valid studies with 90% concordance. Table 1 lists the type of respiratory events noted on scoring. 

Participants with ≥10 apneas/hypopneas per hour in bed on Type 3 home polygraphy (Embletta^®^ Gold™; Embla Systems LLC, Amsterdam, the Netherlands) and symptoms consistent with OSAS were invited to participate in the CPAP-trial. Ability to give informed consent and comply with the protocol (participant or welfare guardian/attorney consent, as appropriate) was necessary. Exclusion criteria were previous exposure to CPAP therapy, arterial oxygen saturation <90% on room air, a history of chronic heart failure or recent myocardial infarction, known moderate or severe dementia, severe behavioural problems that would preclude sleep studies or CPAP treatment, inability to comply with the protocol.

**Figure 1:**
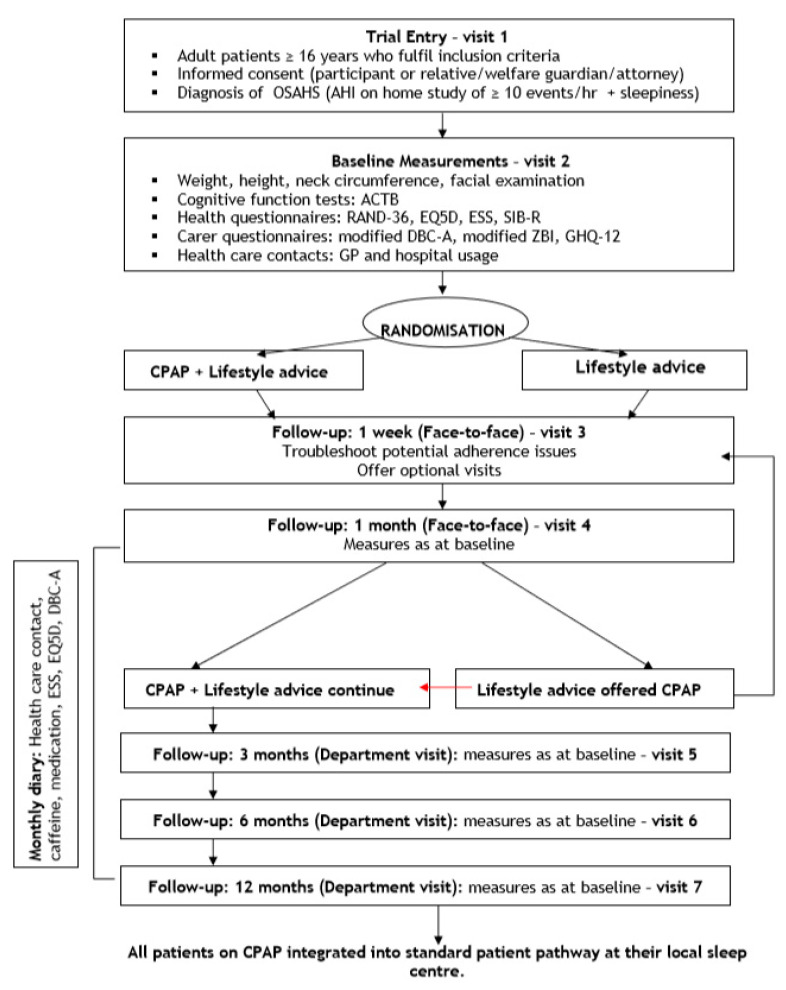
Flow diagram of CPAP evaluation phase of study: trial entry, randomisation, treatment arms, and follow-up schedule.

Figure 1 summarizes the steps in the study. After discussing the results of the home sleep study and with baseline assessments undertaken, participants were randomized by a blinded investigator using balanced block design to CPAP treatment with conservative lifestyle advice or conservative lifestyle advice alone. The latter comprised written advice on diet, exercise, sleep hygiene and sleeping position only. In the CPAP and lifestyle arm, CPAP therapy was initiated and monitored by an unblinded, experienced research nurse. Auto-titrating CPAP devices were used (S8 AutoSet Spirit II™; ResMed (UK) Ltd., Abingdon, UK). All participants received a patient folder containing diaries to complete monthly for the duration of the study and to document any side-effects or difficulties with treatment. Control participants (lifestyle only) were offered CPAP-treatment after 1 month, with additional follow-up at 1-month post-initiation (visit 4 was incorporated to ensure the same follow-up input as for the group initiated on CPAP at randomization). All study participants were reassessed at 3, 6, and 12 months. The CPAP machines were downloaded to a personal computer and information on usage, mask to face time and residual apnoeic events were recorded using the inbuilt logging systems. At the end of the 12-month follow-up visit, all participants using CPAP were integrated into a standard clinical care pathway at their geographically closest sleep service. 

### 2.1. Study-Specific Measures

Height, weight, neck circumference and craniofacial features (presence of macroglossia, gothic palate, adenoidal facies, malocclusion, and tonsillar enlargement) were assessed using standardised techniques [1]. Data obtained on the sleep studies were also recorded. 

### 2.2. Cognitive Function Tests

Participants completed cognitive function tests at baseline, one month, three months, and six months. Sleepiness, behaviour, cognitive function and general health were measured at the same time on each visit at the participant’s home to minimize circadian effects on performance [19]. The primary outcome, subjective sleepiness, was assessed using the pictorial Epworth Sleepiness Scale (pESS) [16]. Cognitive and adaptive function were assessed using the Arizona Cognitive Test Battery (ACTB) [20]: CANTAB Paired Associates Learning (PAL) and Simple Reaction Time (SRT) (Cambridge Cognition Ltd., Cambridge, UK); Modified Dots Task (Frogs and Cats; Down Syndrome Research Group, University of Arizona); Kaufmann Brief Intelligence Test (KBIT-2; Pearson Clinical Assessment, San Antonio, TX, USA); and carer-rated Scales of Independent Behaviour Revised (SIB-R; Riverside Publishing Company, Rolling Meadows, IL, USA). Unfortunately, some data for the Modified Dots task was overwritten due to operator error, and no baseline data were available; data were recovered for the majority of patients for visits 4 and 7 only. All tests were administered by a single researcher who remained blinded to allocation status, CPAP-use and any personal information at all times. 

General health was assessed using the EQ-5D [21] and RAND SF-36 [22]. The printed RAND SF-36 issued to participants in the early stages of the study omitted question 28 in error, resulting in missing data for the majority of participants and rendering outcomes for this domain invalid. Relatives/carers were also asked to complete questionnaires at baseline, 1 month, 3 months, 6 months and 12 months in addition to open-ended qualitative questions about their experiences of caring [23]. Behavioural and emotional disturbance were assessed using the Developmental Behaviour Checklist for Adults subscales (DBC-A) for disruptive behaviour, anxiety/antisocial behaviour and depressive behaviour [24]. Additional questionnaires included the General Health Questionnaire (GHQ-12) [25], the pESS [16] with relatives/carers independently rating the participant’s sleepiness.

### 2.3. Monthly Diary

Participants and relatives/carers were asked to fill out a diary during the 12-month period. This was used to record GP and hospital visits, caffeine intake, medication and CPAP side-effects. All questionnaires were filled in at each visit by participants assisted by a relative/carer as appropriate to allow for objective evaluation. The same individual was required to fill in the questionnaires throughout the study to maintain internal consistency.

### 2.4. Statistical Analysis

Based on a questionnaire study we conducted in over 5000 adults with DS [15], we expected over 100 people to respond to an invitation to participate in the CPAP trial. Due to unreasonable delays by the local ethics committee and funding constraints, this recruitment rate was not achieved in the time period of the study (2011–2015). 

A primary analysis by ‘intention to treat’ was initially planned with a second ‘per-protocol’ analysis excluding participants who had abandoned CPAP therapy during the pilot, randomised controlled trial. The intention to treat analysis was deployed for the first part of the study (randomised, parallel controlled trial at 1 month) only. Based on a previous study conducted in our department, in patients from the OSAHS general population [26], the proposed sample size was calculated to provide 90% power to show large differences of 0.8 SD between treatment groups (using general health indices and the pESS) in the randomised, pilot trial. In order to achieve a power of 80% we aimed to recruit a minimum of 26 participants into each arm of the study. Since there was no published data at the time on adherence rates to CPAP therapy in adults with DS, we made allowance for a drop-out rate of 10% based on previous studies. Since the adult DS population is finite, we were willing to accept that any dropouts would lead to a small decrement in study power. 

Statistical analyses were conducted using SPSS Statistics version 19 (IBM Corp, Armonk, NY, USA). All analyses were two-tailed, with significance set at *p* = 0.001 due to multiple testing. All variables were normality-checked. Discrete variables were evaluated using the Chi-square test and continuous variables using Student’s *t*-test. Pearson’s and Spearman’s rank correlations were used to explore correlations between parametric and non-parametric variables respectively. The Mann-Whitney U test was used for non-parametric variables. Binary logistic regression (categorical variables), generalized linear modelling (continuous variables) and multivariate regression were undertaken to explore associations between the variable/s of interest and relevant independent and dependent factors. These analyses were used to explore predictors of CPAP-use and the effects of total hours of CPAP-use over 12 months on changes in behaviour, KBIT-scores and the pESS. Results are presented as mean ± standard deviation for parametric variables, median with interquartile range (IQR 25–75%) for non-parametric data, or as number and percentage. One-month assessments were analysed as a randomized, controlled trial of CPAP therapy versus conservative lifestyle measures. Thereafter, individuals in the lifestyle group commenced CPAP and all study participants were pooled into a single group and analysed as a prospective cohort study. Data were entered blind to randomization for the pilot study. At the end of 12 months, all data were locked, and analysis was undertaken by a researcher blinded to CPAP adherence and any additional personal information. 

## 3. Results

**Figure 2:**
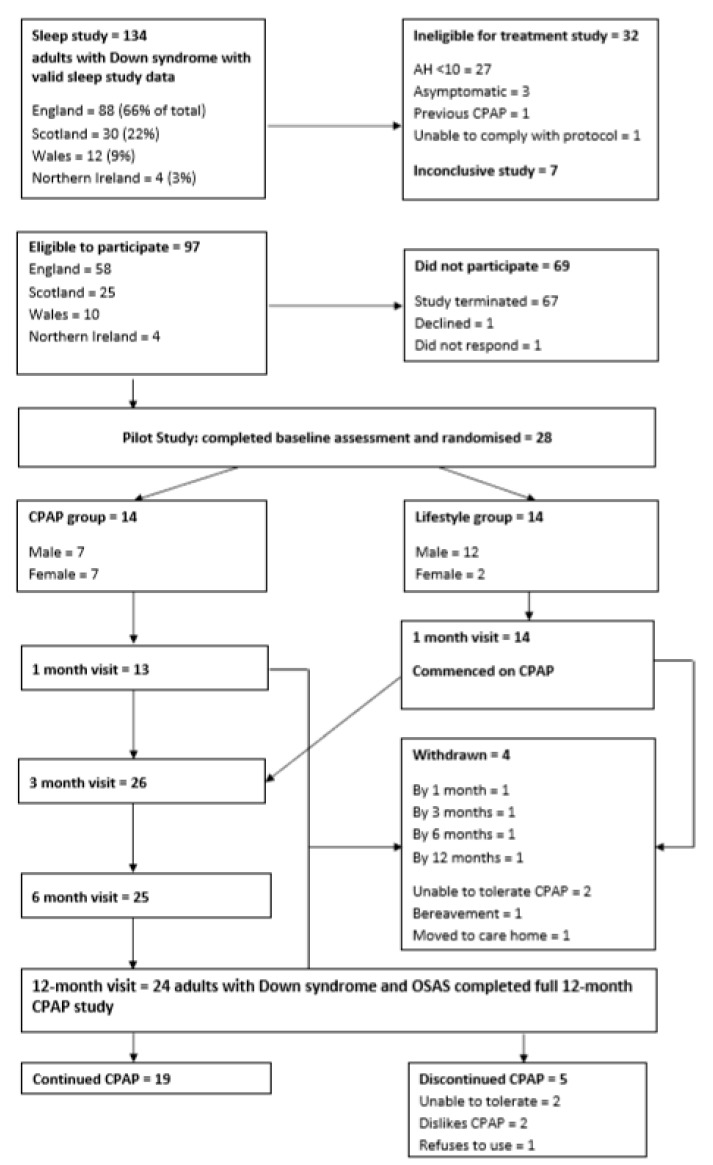
CONSORT diagram summarising enrolment in and completion of the treatment phase of the study.

Figure 2 summarises participation data. Of 97 eligible participants, 28 (19 males; nine females) were enrolled and randomised. Twenty-four participants completed the full 12-month study. Of this group, one individual withdrew prior to 12 months due to family bereavement but completed and returned participant and caregiver questionnaires by post at 12 months. In the event of participant withdrawal, the CPAP machine was retrieved and downloaded, allowing compliance data to be obtained up to the time of withdrawal from the study. 

### 3.1. Participant Characteristics

No significant differences in anthropometric data by sex were saw Table 1. Most participants (71%) reported a Trisomy 21 karyotype and 7% reported mosaicism; 21% did not know their karyotype. Participants had moderate (64%) to severe (36%) intellectual disability. Eighty-nine percent lived at home with parent(s), the remaining 11% lived in supported accommodation. The mean age of participants was 28 ± 9 years. Twenty-six percent had a normal BMI; the remainder were overweight or obese. Mean BMI was 31.5 ± 7.9 kg/m^2^, with a higher mean BMI in females (37.4 ± 6.9 kg/m^2^_;_ *p* = 0.009). BMI and collar size did not change over the 12-month period. 

### 3.2. Randomized, Controlled Pilot Trial of CPAP Therapy versus Conservative Lifestyle Measures 

At one-month post-randomisation, no significant between-group differences were observed (data not shown). Actual CPAP use by the treatment group at 1-month averaged 36 ± 9 days from CPAP initiation with 35.7% (0.0–52.6%) of used days averaging ≥4 h usage, the conventionally accepted therapeutic minimum in the general population [27]. The mean 95th centile pressure was 8.9 ± 2.8 cmH_2_O, with a mean leak within normal limits (0.3 L/s (0.2–0.4) L/s). Pressure, leak, and AHI data were unavailable for one participant due to machine error. 

### 3.3. Prospective Treatment Trial

The lifestyle group did not change significantly between baseline and one-month visits (pre-CPAP). Therefore, the lifestyle group baseline measures were pooled with the CPAP group baseline measures for use in the whole group prospective, treatment study analysis. Sleepiness, behaviour, cognitive function, and general health outcomes for the whole group on CPAP over 12 months are summarized in Table 2 and Table 3.

### 3.4. Subjective Sleepiness

Mean self-rated pESS scores improved significantly across the 12 months, from 11 ± 6/24 at baseline to 7 ± 6/24 at 3 months (*p* < 0.0001), and 6 ± 5/24 at 12 months (*p* = 0.001). There was an overall downward trend in proxy-rated pESS scores across the 12 months on CPAP, although this did not reach significance. A reduction in pESS scores was significantly associated with greater hours of CPAP use over 12 months (B = −0.003; *p* = 0.039; CI95% −0.005–0.0001)

### 3.5. Cognitive and Adaptive Function

KBIT-2 verbal scores showed significant improvement at 12 months (*p* = 0.001), with a trend towards improvement appearing at three months (*p* = 0.002). Mean baseline verbal score was 31 ± 15, rising to 37 ± 19 at 12 months. Non-verbal subscale scores increased at three and 12 months but did not reach statistical significance (*p* = 0.05, *p* = 0.01 respectively). The mean baseline score in this subscale was 13 ± 5, rising to 20 ± 17 at 12 months. Individual performance on the KBIT-2 verbal and non-verbal scores are shown in Appendix A. 

### 3.6. Behavioural and Emotional Disturbances 

A significant decrease in DBC-A Disruptive subscale scores was observed at 12 months in comparison to baseline, indicating improvement in Disruptive behaviour (1(0–3) v. 4 (2–8); *p* < 0.0001), and in severity and breadth of behavioural/emotional disturbance (both *p* < 0.0001). Similar reductions were also noted in the three Depressive behaviour scores (all *p* = 0.001). No significant change was evident in Anxiety/Antisocial behaviour scores, though a floor effect was noted. The change in Disruptive behaviour scores exhibited a steady reduction from baseline, reaching significance at three months (*p* = 0.001) which was maintained at 12 months (*p* < 0.0001). Regression analysis revealed that only depressive scores were reduced significantly with total hours of CPAP use over 12 months (B = −0.001; *p* = 0.026; CI95% −0.003–0.0001). Improvement in the disruptive scores was related to improvement in depression only (B = −1.8; *p* < 0.0001; CI95% 0.42–1.2) suggesting significant collinearity in these scores.

Individual changes on all 3 DBC-A subscales are shown in Appendix B. 

### 3.7. General Health Measures

The reported EQ-5D visual analogue scale was 81 ± 19% overall at baseline; this did not differ significantly over the course of the study, despite CPAP-use. Reported problems with mobility, self-care, carrying out usual activities, pain/discomfort and anxiety/depression were all low at baseline, and did not vary over the 12 months.

General health increased from as score of 74 ± 23% at baseline to 84 ± 21% at 12 months but was not considered statistically significant (*p* = 0.02 at 3 months, *p* = 0.05 at 12 months).

No significant changes were evident in physical or emotional role limitation, energy/fatigue, emotional wellbeing, social functioning or pain as measured using the RAND SF-36. Notably, energy/fatigue scores were low at baseline, with a mean score of 50 ± 21%. This rose to 59 ± 20% after 12 months on CPAP (*p* = 0.14). The low number of participants with RAND SF-36 emotional wellbeing scores should be noted (*n* = 5 at baseline and 3 months, and *n* = 4 at 12 months). 

However, physical functioning increased significantly from baseline (68 ± 31%) to three months (73 ± 30%; *p* < 0.0001); further increases at 12 months (78 ± 27%) did not reach statistical significance (*p* = 0.03).

### 3.8. CPAP Compliance over Time

At 1-month post-CPAP initiation (mean duration from initiation = 39 ± 4 days), CPAP adherence (*n* = 27) was generally low, with a mean usage of 1.7 h/night (0.2–4.5) h/night and a median usage of 2.87 h/night (1.1–6.7) h/night. CPAP was used on 55 ± 35% of days; the mean proportion of nights on which CPAP was used for ≥4 h was 35.7% (0.0–84.0%). The mean total usage was 64.6 h (8.2–143.3 h). Mean 95th centile pressure was 8.2 ± 3.0 cmH_2_O and 95th centile leak fell within normal limits at 0.3 L/min (0.2–0.5) L/min. The mean machine-derived AHI was 9.7 h (1.2–11.8)/h in bed).

No significant changes were noted with respect to adherence, pressure, leak or AHI at 3, 6, or 12 months post-CPAP initiation. Mean adherence at 12 months for 25 participants (352 ± 17 days from CPAP initiation) remained low: mean usage 1.5 h/night (0.3–4.6) h/night; median usage 2.3 h/night (01.0–6.4) h/night; days used 53.7 ± 33.7%; duration ≥4 h 34.3% (4.1–83.3%). The mean 95th centile pressure was 8.6 ± 2.2 cmH_2_O, and the 95th centile leak was 0.3 ± 0.2 L/min. The mean AHI was 6.7/h in bed (2.9–8.7/h). 

Regression analysis did not reveal any significant associations between age, sex, BMI, AHI, oxygen desaturation index, behaviour, or sleepiness at baseline with CPAP compliance at 12 months (Appendix C).

### 3.9. Acceptability of CPAP

All participants were fitted with full face masks, eight of whom required a mask change during the trial. At 1 month, one participant reported mask-related skin irritation and another participant reported anxiety related to the CPAP equipment. One participant withdrew due to inability to tolerate CPAP. At three months, a further participant had withdrawn from the study due to CPAP-intolerance. By 12 months, 24 participants remained in the study, with two participants having withdrawn due to family bereavement, and moving into residential care, respectively. At the end of the study, 6 (25%) participants reported some adverse events related to CPAP-use: anxiety (8%); intolerance (8%); skin irritation (4%); wakening due to CPAP (4%). However, nineteen individuals elected to continue CPAP at study completion. 

Humidification was commenced in response to reported symptoms (e.g., nasal congestion, dryness) using the unblinded nurse’s clinical judgement. Two individuals (7%) used humidification at one month, four (15%) at three months, seven (28%) at six months, and seven (29%) at 12 months. No significant differences were evident in CPAP outcomes between those using humidification and those without (data not shown).

## 4. Discussion

This is the first randomized, controlled pilot study of CPAP therapy in adults with Down syndrome and to date, the longest prospective study of CPAP in adults with DS. The reason for offering controls in the pilot study the intervention at one-month post-randomisation was based on short studies undertaken in the general population that have shown improvements after 4 weeks of CPAP-use [28,29,30]. However, when designing the study, we did not factor in quite as many issues with CPAP and mask-use as we experienced; this was unknown to us at the time. Despite the small number of participants, real and significant improvements in sleepiness, behaviour, and daytime cognitive function were demonstrated at 12 months whilst using CPAP. 

To date, no other studies have systematically evaluated effectiveness and acceptability of CPAP in the community-dwelling, DS population. Trois et al. reported in passing on the treatment of adult DS individuals with OSA presenting to a sleep centre [31]. Nine of the 14 adults referred for CPAP treatment were followed up by the researchers, one sought treatment elsewhere, and four participants were lost to follow-up. CPAP was acceptable to the majority, with over half using therapy for 6–8 h/night; their families reported subjective improvements in sleepiness and daytime function. Two individuals did not accept CPAP, one quit due to side-effects, and one used CPAP sub-maximally; these were similar results to ours. However, no formal assessment of sleepiness, cognition or behaviour was undertaken, and no standard method for CPAP initiation and support was described. 

There is some evidence, albeit not firmly conclusive, that CPAP improves cognitive function in the general population with OSAS [9,10,11], but little is known about its impact in the Down syndrome population. A study of the effects of CPAP on neurobehavioral outcomes in 52 children with OSAS included 10 individuals with developmental delays, 6 of whom had Down syndrome [32]. Significant improvements in ESS, behaviour, and quality of life were reported, but the very small sample size (*n* = 6) limits generalization of findings.

The AH requirement for entry to the treatment phase of this study was AH ≥ 10/h in bed. General population studies have assessed the efficacy of treating mild OSAS with CPAP, with two placebo-controlled studies of symptomatic individuals with an AHI in the mild range (5.0–14.9 events per hour) demonstrating improvements in sleepiness, mood and daytime function, even with low CPAP compliance [28,33]. Adults with Down syndrome may be more sensitive to the cognitive effects of untreated OSAS given the existing cognitive impairment seen in this group, and so may stand to benefit even when diagnosed with mild OSAS [34].

Similar studies in other neurological disorders, as well as in the older general population have also defined lower AHI thresholds to account for possible differences in the aetiology of the sleep disordered breathing and to minimise Type I error [33,35,36].

## 5. Conclusions

Despite small participant numbers, we demonstrated that CPAP-use lead to improvements in subjective sleepiness, behavioural and emotional outcomes and cognitive function in a group of community-dwelling individuals with DS and OSAS exhibiting moderate/severe intellectual disability. Cognitive impairment and behavioural problems seen in adults with DS may be compounded by untreated OSAS. Given the potential benefits in terms of improved daytime function and quality of life, a further, larger-scale, randomized, controlled trial of CPAP in this population is warranted.

### Limitations and Future Research

There are a number of limitations to this study. The study is small in size, unfortunately cut short due to unacceptable delays in ethical approval which appear to be common for research in individuals with intellectual disability, resulting in insufficient funding for the necessary duration of the study [37,38]. However, it remains one of the largest, systematic studies of CPAP in community-dwelling adults with DS worldwide, albeit underpowered (sample size 24). Despite the sample size and our conservative significance threshold, marked improvements in sleepiness and scores of depression were demonstrated. These positive results, alongside trends towards improvement in other domains, suggest that a larger, multicentre trial is of merit. 

No significant differences in outcomes between the two groups were evident after the one- month randomised phase of the study. One month was selected as this length of time has been sufficient to show a significant change in the general population [28,29,30], although other studies in the general population have used longer periods up to 12 months [33]. Having now demonstrated that statistically significant change occurs across a wide range of behavioural and intellectual parameters, further studies could safely be randomised to three months of best supportive care only for the control group. Problems with mask fit and comfort were encountered during this study, with many commercially available CPAP interfaces proving to be too large for participants, even in the smallest sizes. All participants were fitted with full-face masks due to obligate mouth breathing, on account of relative macroglossia and low tone. Masks designed for individuals with DS, taking into account the midface hypoplasia and short philtrum which is common in this group, may be required, and recent advances in 3D printing technology may allow personalised masks modelled on each individual’s face to become an option in the very near future. 

It is our usual clinical practice to introduce heated humidification only as required due to CPAP side-effects such as nasal congestion or dryness, and so a similar approach was taken in this study. With hindsight, given the issues of increased mucus secretion and frequent respiratory tract infections in individuals with DS, it may have been appropriate to start all participants on CPAP with humidification from the outset. There is conflicting evidence as to whether humidification improves CPAP compliance, with some studies demonstrating significant improvement [39] and others not [40]. Although some participants did report side-effects and were later issued with humidification, it is possible that this contributed to reduced compliance. A study of fixed versus auto-titrating CPAP in the general population noted a significant order effect, with neither type of device improving compliance but participants preferring whichever they used first [41]; it is possible that a similar effect could be ascribed to the use of humidification. 

Given the small sample size, it was not viable to undertake the planned health economic analysis. Health economic benefits of CPAP-use have been demonstrated in older adults in the general population, despite modest compliance [33], and should be studied in adults with DS.

Changes were noted in pESS, KBIT-2 scores and DBC-A scores despite a low median usage of CPAP across the group, and it would be of interest to assess whether these measures would improve in a dose-dependent manner. Although steps were taken to encourage CPAP usage, future studies might be able to employ additional strategies to increase adherence. Our group of DS adults were all community-dwelling, resident throughout the United Kingdom and at the time of this study, telemonitoring was not being deployed routinely across all sleep centres. Cognitive behavioural interventions, self-management, peer support, intensive education and follow-up have all been shown to improve CPAP compliance in the general population [42,43]. Incentivization via token economy has been shown to increase adherence to physical activity programmes in individuals with Down syndrome [44], offering many avenues to explore in the future. All in all, this study demonstrated that the majority of adults with DS can tolerate CPAP, with 80% of those entering the study continuing after 12 months; its use as a standard treatment for OSAS in this group should not be ruled out.

## Figures and Tables

**Table 1:** Characteristics of randomisation groups at baseline—pre-CPAP (mean ± SD or median (IQR) as appropriate). CPAP: continuous positive airway pressure.

Anthropometric, Behavioural, and Sleep Characteristics	Total Included	Lifestyle		CPAP		*p*
		*n* = 14		*n* = 14		
Gender (males: females)	28	12:2		7:7		0.10
Age (years)	28	27 ± 8		29 ± 10		0.54
Body Mass Index (kg/m^2^)	27	30.0 ± 7.4		33.2 ± 8.3		0.29
Collar size (cm)	28	41.4 ± 4.2		41.6 ± 5.7		0.93
Karyotype						
Trisomy 21	28	10	71.4%	10	71.4%	0.48
Translocation		0	0.0%	0	0.0%	
Mosaic		2	14.3%	0	0.0%	
Unknown/not tested		2	14.3%	4	28.6%	
Level of intellectual disability:						
Mild	28	0	0.0%	0	0.0%	0.70
Moderate		10	71.4%	8	57.1%	
Severe		4	28.6%	6	42.9%	
Profound		0	0.0%	0	0.0%	
Living arrangements:						
At home with parents	28	12	85.7%	13	92.9%	1.00
Supported accommodation		2	14.3%	1	7.1%	
Malocclusion:						
A	28	2	14.3%	3	21.4%	0.88
B		4	28.6%	4	28.6%	
C		0	0.0%	0	0.0%	
D		8	57.1%	7	50.0%	
Macroglossia	28	4	28.6%	5	35.7%	1.00
Gothic palate	28	13	92.9%	13	92.9%	1.00
Adenoidal facies	28	3	21.4%	3	21.4%	1.00
Mallampati Score:						
Class I	28	1	7.1%	0	0.0%	0.18
Class II		1	7.1%	5	35.7%	
Class III		7	50.0%	7	50.0%	
Class IV		5	35.7%	2	14.3%	
Pictorial Epworth Sleepiness Scale (pESS):						
Self-rated	27	10 ± 5		11 ± 7		0.70
Carer-rated	20	10 ± 4		12 ± 6		0.36
Developmental Behaviour Checklist for Adults (DBC-A):						
Disruptive behaviour subscale (scale range 0–34)	28	5 (3–8)		4 (2–9)		0.84
Anxiety/Antisocial subscale (scale range −2–14)	28	1 (−1–1)		0 (0–1)		0.87
Depressive subscale (scale range 0–18)	28	2 (1–4)		1 (0–4)		0.73
Kaufmann Brief Intelligence Test (KBIT-2):						
Raw score verbal	28	30.6 ± 17.6		31.9 ± 13.3		0.83
Raw score non-verbal	28	12.3 ± 6.3		14.4 ± 4.2		0.30
Scales of Independent Behaviour—Revised (SIB-R) Adaptive Behaviour Short Form:						
Raw score	27	84.2 ± 14.6		80.1 ± 11.1		0.50
CANTAB Paired Associates Learning (PAL):						
Mean errors to success	27	4.5 ± 3.5		5.1 ± 2.9		0.62
First trial memory score	28	11.4 ± 7.4		9.9 ± 4.3		0.52
CANTAL Simple Reaction Time (SRT):						
Mean correct latency (ms)	28	606.8 ± 346.2		557.3 ± 311.8		0.69
EQ-5D:						
Mobility	28	0.0 (0.0–1.3)		0.0 (0.0–2.0)		0.60
Self-care	28	0.0 (0.0–1.3)		0.0 (0.0–1.3)		0.95
Usual activities	28	0.0 (0.0–2.0)		0.0 (0.0–1.3)		0.80
Pain/discomfort	28	1.0 (0.0–1.0)		0.0 (0.0–2.0)		0.84
Anxiety/depression	28	0.0 (0.0–1.0)		0.0 (0.0–1.0)		0.70
Health state (visual analogue scale; %)	27	80 (65–90)		93 (75–100)		0.08
RAND SF-36:						
Total percent	28	80.8 (63.2–85.0)		63.1 (40.1–81.7)		0.13
Physical functioning	27	90.0 (45.0–96.3)		65.0 (30.0–97.5)		0.26
Role limitations due to physical health	28	100.00 (100.0–100.0)		100.0 (18.8–100.0)		0.19
Role limitations due to emotional problems	28	100.0 (100.0–100.0)		100 (83.3–100.0)		0.73
Energy/fatigue	28	62.5 (48.8–65.0)		47.5 (32.5–55.0)		0.13
Emotional well-being	5	84.1 (76.2–84.1)		60.0 (32.0–60.0)		0.20
Social functioning	27	100.0 (71.9–100.0)		87.5 (68.8–100.0)		0.38
Pain	28	50.0 (49.4–90.0)		50.0 (40.6–80.0)		0.27
General health	28	80.0 (62.5–95.0)		75.0 (47.5–95.0)		0.76
General Health Questionnaire 12-item version (GHQ12):						
Total score	27	10.7 ± 2.5		10.5 ± 3.9		0.84
Modified carer burden inventory (CBI):						
Total score	27	10.8 ± 7.0		9.0 ± 6.7		0.51
Objective sleep study variables on home-based polygraphy:						
Total recording time (min)	25	525.5 ± 102.6		475.2 ± 104.9		0.24
Apnoeas/Hypopnoeas per hour in bed	24	25.0 (15.8–46.5)		31.1 (14.1–50.1)		0.78
Supine	24	27.3 (17.2–46.4)		50.1 (16.9–97.3)		0.28
Non-supine	24	17.7 (6.0–24.3)		20.1 (7.4–36.8)		0.39
Obstructive	24	4.0 (1.1–9.9)		3.4 (1.2–23.7)		0.73
Central	24	0.2 (0.0–0.7)		3.9 (0.0–2.2)		0.277
Mixed	24	0.0 (0.0–0.2)		0.0 (0.0–0.0)		0.42
Hypopnoea	24	21.9 (11.9–30.5)		22.7 (12.2–33.0)		1.00
Mean SpO2 (%)	24	93.3 ± 4.2		94.4 ± 2.3		0.42
SpO2 nadir (%)	24	81.3 ± 8.9		80.7 ± 9.2		0.88
Average desaturation (%)	24	5.0 ± 0.8		6.0 ± 1.4		0.04
Oxygen desaturation index per hour in bed	23	7.5 (2.1–23.7)		7.3 (1.6–21.9)		1.00

**Table 2:** Comparison of whole group at baseline and 12 months (mean ± SD or median (IQR) as appropriate).

	Baseline Visit		12 Month Visit		
	*n* = 28		*n* = 24		
Participant Characteristics	Total Included	Value	Total Included	Value	*p*
Body Mass Index (kg/m^2^)	26	31.0 ± 7.5	23	32.5 ± 7.3	0.16
Collar size (cm)	27	41.3 ± 4.9	24	41.7 ± 5.3	1.00
Developmental Behaviour Checklist for Adults (DBC-A):					
Disruptive behaviour subscale (scale range 0–34)	28	4 (2–8)	23	1 (0–3)	<0.0001
Anxiety/Antisocial subscale (scale range −2–14)	28	0 (−1–1)	23	0 (−1–0)	0.03
Depressive subscale (scale range 0–18)	28	1 (1–4)	23	0 (0–1)	0.001
Pictorial Epworth Sleepiness Scale (pESS):					
Self-rated	27	11 ± 6	24	6 ± 5	0.001
Carer-rated	20	11 ± 5	16	7 ± 5	0.03
Kaufmann Brief Intelligence Test (KBIT-2):					
Raw score verbal	28	31.3 ± 15.3	24	37.4 ± 18.6	0.001
Raw score non-verbal	28	13.4 ± 5.4	24	19.5 ± 17.1	0.01
Scales of Independent Behaviour—Revised (SIB-R) Adaptive Behavior Short Form:					
Raw score	27	82.6 ± 12.9	22	85.4 ± 12.5	0.33
CANTAB Paired Associates Learning (PAL):					
Mean errors to success	27	4.9 ± 3.2	22	4.5 ± 3.8	0.44
First trial memory score	28	10.6 ± 6.0	24	12.0 ± 6.6	0.24
CANTAL Simple Reaction Time (SRT):					
Mean correct latency (ms)	28	582.1 ± 324.3	24	599.1 ± 289.9	0.43

**Table 3:** Change (Δ) from baseline in whole group at 1, 3, 6, and 12 months on CPAP (mean ± SD or median (IQR) as appropriate).

	1 Month Post-CPAP Initiation		3 Month Visit		6 Month Visit				12 Month Visit		
	*n* = 27		*n* = 26		*n* = 26 *				*n* = 25 *		
Participant characteristics	Total included	Δ from baseline	*p*	Total included	Δ from baseline	*p*	Total included	Δ from baseline	*p*	Total included	Δ from baseline
Developmental Behaviour Checklist for Adults (DBC-A):											
Disruptive behaviour subscale (scale range 0–34)	26	−1.38 ± 2.61	0.01	26	−2.65 ± 3.65	0.001	24	−2.50 ± 3.43	0.002	23	−3.13 ± 3.11
Anxiety/Antisocial subscale (scale range −2–14)	26	−0.58 ± 1.30	0.03	26	−0.54 ± 1.29	0.05	24	−0.42 ± 1.14	0.09	23	−0.48 ± 0.95
Depressive subscale (scale range 0–18)	26	−0.96 ± 2.24	0.04	26	−1.65 ± 2.94	0.01	24	−1.58 ± 2.87	0.01	23	−1.65 ± 2.66
Pictorial Epworth Sleepiness Scale (pESS):											
Self-rated	26	−1.27 ± 5.94	0.29	25	−4.36 ± 5.37	<0.0001	24	−4.75 ± 5.61	<0.0001	24	−4.75 ± 6.23
Carer-rated	21	−1.33 ± 5.58	0.29	19	−3.00 ± 4.97	0.02	17	−4.29 ± 6.57	0.02	16	−3.44 ± 5.70
Kaufmann Brief Intelligence Test (KBIT-2)											
Raw score verbal	25	3.20 ± 5.45	0.01	26	4.04 ± 5.99	0.002	25	3.88 ± 6.55	0.01	24	4.42 ± 5.88
Raw score non-verbal	25	5.16 ± 15.21	0.10	26	4.92 ± 12.15	0.05	25	4.92 ± 12.08	0.05	24	6.13 ± 14.79
Continuous positive airway pressure (CPAP):	Total included	Raw value	*p*	Total included	Δ from 1 month	*p*	Total included	Δ from 1 month	*p*	Total included	Δ from 1 month
Total days since CPAP initiation	-	34 ± 9	-	26	41.77 ± 20.76	-	26	127.81 ± 20.03	-	25	318.48 ± 17.11
Days used (%)	-	55.0 ± 34.5	-	26	−1.27 ± 12.3	0.61	26	0.10 ± 15.72	0.98	25	−3.80 ± 15.34
Days used ≥ 4 h (%)	-	35.7 (0.0–84.0)	-	26	−0.56 ± 6.43	0.66	26	1.70 ± 11.4	0.45	25	0.76 ± 15.96
Total usage (h)	-	64.6 (8.2–143.3)	-	26	117.01 ± 147.14	-	26	358.3 ± 365.07	-	25	812.74 ± 808.27
Mean usage (h)	-	1.7 (0.2–4.5)	-	26	−0.19 ± 1.15	0.41	26	−0.07 ± 1.21	0.79	25	−0.34 ± 1.25
Median usage (h)	-	2.8 (1.1–6.7)	-	26	−0.04 ± 0.46	0.68	26	0.10 ± 0.76	0.51	25	0.04 ± 1.04
95th centile pressure (cmH_2_O)	-	8.2 ± 3.0	-	26	0.12 ± 1.4	-	26	0.16 ± 0.14	-	25	0.58 ± 1.79
95th centile leak (L/s)	-	0.3 (0.2–0.5)	-	25 **	−0.04 ± 0.26	0.50	25 **	−0.12 ± 0.40	0.16	24 **	−0.20 ± 0.48
Apnoea/Hypopnoea index (derived from CPAP machine)	-	9.7 (1.2–11.8)	-	25 **	−0.23 ± 1.8	0.54	25 **	−0.66 ± 2.46	0.19	24 **	−1.10 ± 2.73

* Includes CPAP data from machine download of patient at withdrawal. ** No data due to machine error.

**Figure A1:**
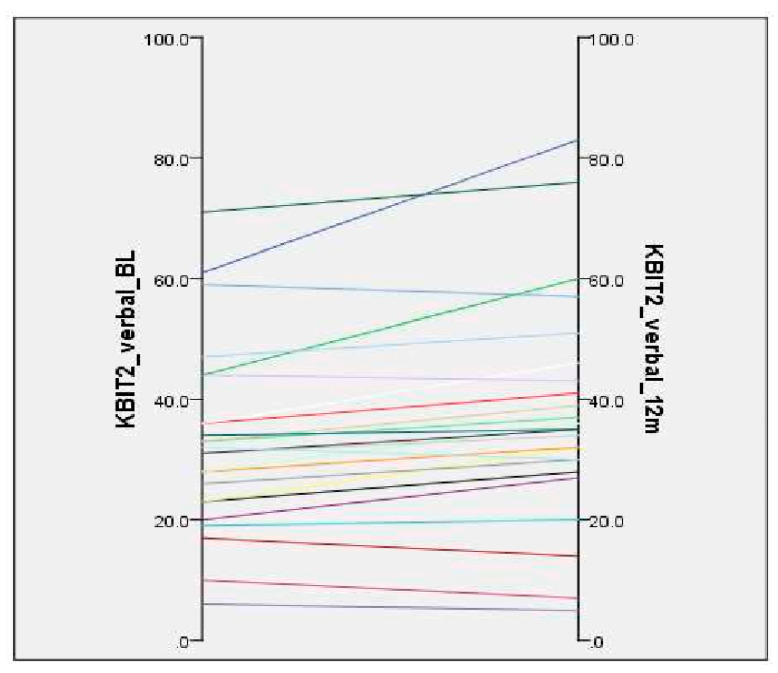
Change in K-Bit Verbal Scores over 12 months.

**Figure A2:**
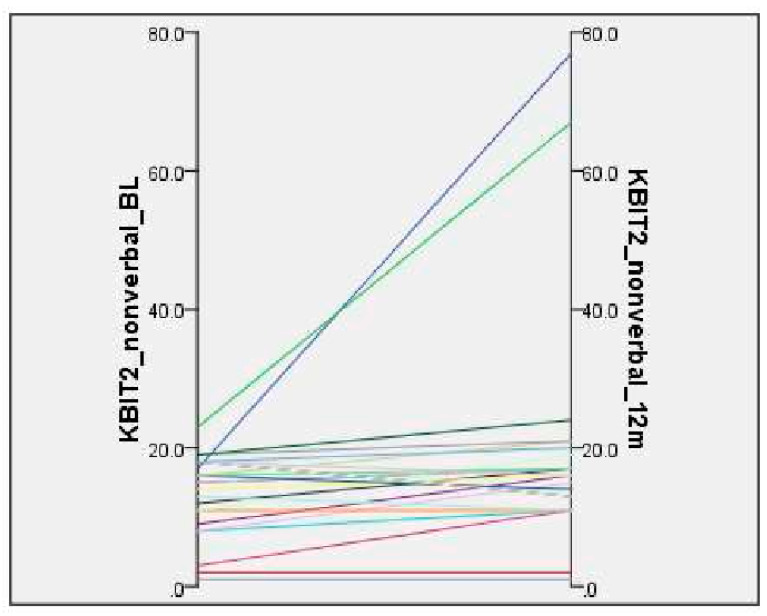
Change in KBIT2 non-verbal scores at 12 months from baseline.

**Figure A3:**
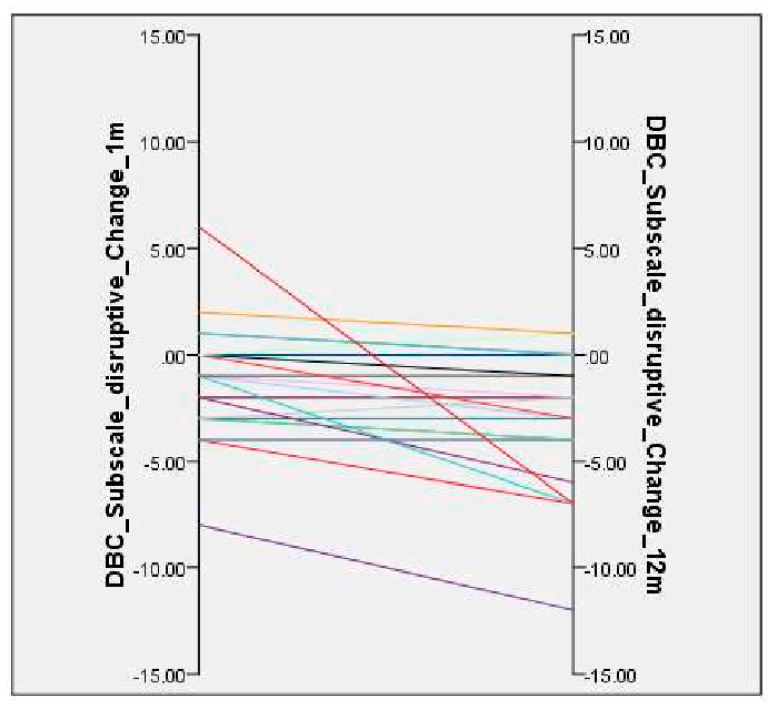
Individual scores on the Developmental Behaviour Checklist for Adults (DBC-A) subscales at baseline and at 12 months: Disruptive Behaviour Scale.

**Figure A4:**
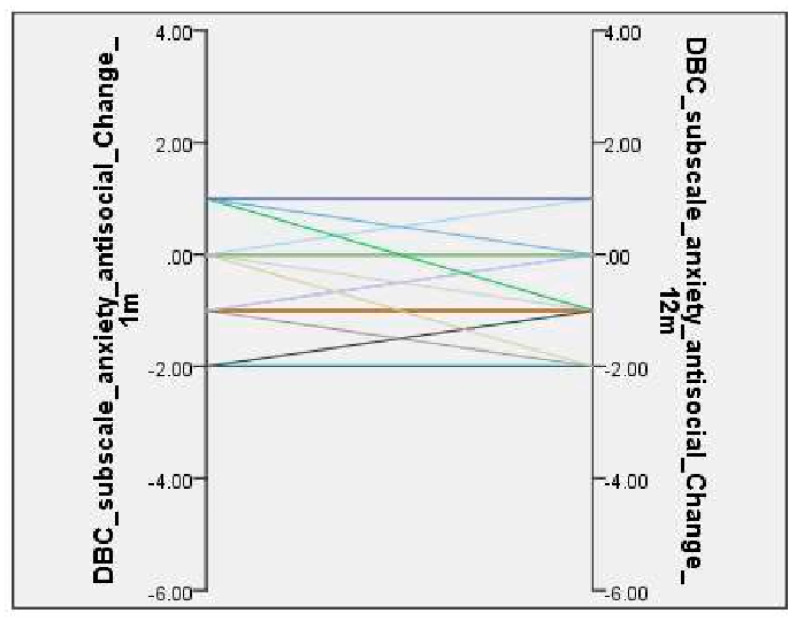
Scores on the Developmental Behaviour Checklist for Adults (DBC-A) subscales at baseline and at 12 months: Anxiety/Antisocial Scale.

**Figure A5:**
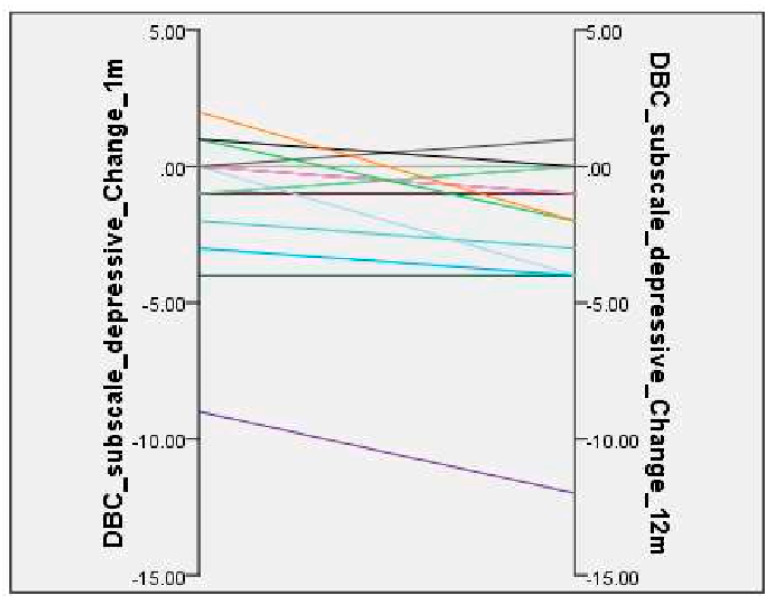
Individual scores on the Developmental Behaviour Checklist for Adults (DBC-A) subscales at baseline and at 12 months: Depressive Scale.

## Appendix C. Determinants of CPAP Usage at 12 Months Assessed by Generalised Linear Modelling

**Table A1:** Determinants of CPAP usage at 12 m assessed by generalised linear modelling.

Variable	Total Included	Determinants Remaining in Model	Estimate	95% CI Lower	95% CI Upper	*p*
Total usage (h) at 12 m *	24	ID level (moderate; severe)	−942.13	−1774.77	−109.49	0.03
		Age	−38.80	−82.44	4.75	0.08
		Gender	−161.32	−986.60	663.97	0.70
		BMI	−14.00	−62.10	34.10	0.57
		pESS (self-rated)	−83.47	−156.01	−10.93	0.02
Median CPAP usage (h) at 12 m *	24	ID level (moderate; severe)	−3.52	−6.39	−0.64	0.02
		Age	−0.13	−0.29	0.02	0.08
		Gender	−0.65	−3.50	2.20	0.66
		BMI	−0.11	−0.28	0.06	0.19
		pESS (self-rated)	−0.28	−0.53	−0.03	0.03
Median CPAP usage ≥4 h at 12 m **		ID level (moderate; severe)	0.11	0.01	2.31	0.16
		Age	0.87	0.71	1.069	0.19
		Gender	0.42	0.02	10.56	0.60
		BMI	0.88	0.70	1.10	0.26
		pESS (self-rated)	0.73	0.54	0.99	0.04

Determinants of CPAP usage at 12 m assessed by generalised linear modelling * or binary logistic regression ** as appropriate. Estimate reported as β or Exp(B).

## References

[1] World Health Organization. WHO (2015). Genes and Human Disease. http://www.who.int/genomics/public/geneticdiseases/en/index1.html.

[2] Wu J., Morris J.K. (2013). The population prevalence of Down’s syndrome in England and Wales in 2011. Eur. J. Hum. Genet..

[3] Presson A.P., Partyka G., Jensen K.M., Devine O.J., Rasmussen S.A., McCabe L.L., McCabe E.R.B. (2013). Current Estimate of Down Syndrome Population Prevalence in the United States. J. Pediatr..

[4] Heinzer R., Vat S., Marques-Vidal P., Marti-Soler H., Andries D., Tobback N., Mooser V., Preisig M., Malhotra A., Waeber G. (2015). Prevalence of sleep-disordered breathing in the general population: The HypnoLaus study. Lancet Respir. Med..

[5] Bonsignore M.R., Baiamonte P., Mazzuca E., Castrogiovanni A., Marrone O. (2019). Obstructive sleep apnea and comorbidities: A dangerous liaison. Multidiscip. Respir. Med..

[6] Hill E.A. (2016). Obstructive sleep apnoea/hypopnoea syndrome in adults with Down syndrome. Breathe.

[7] Jordan A.S., McSharry D.G., Malhotra A. (2014). Adult obstructive sleep apnoea. Lancet.

[8] Fernandez F., Edgin J.O. (2013). Poor Sleep as a Precursor to Cognitive Decline in Down Syndrome: A Hypothesis. J. Alzheimer’s Dis. Park..

[9] Wallace A., Bucks R.S. (2013). Memory and Obstructive Sleep Apnea: A Meta-Analysis. Sleep.

[10] Patil S.P., Ayappa I.A., Caples S.M., Kimoff R.J., Patel S.R., Harrod C.G. (2019). Treatment of Adult Obstructive Sleep Apnea With Positive Airway Pressure: An American Academy of Sleep Medicine Systematic Review, Meta-Analysis, and GRADE Assessment. J. Clin. Sleep Med..

[11] Labarca G., Dreyse J., Drake L., Jorquera J., Barbe F. (2020). Efficacy of continuous positive airway pressure (CPAP) in the prevention of cardiovascular events in patients with obstructive sleep apnea: Systematic review and meta-analysis. Sleep Med. Rev..

[12] Altintas N., Riha R.L. (2019). Non-sleepy obstructive sleep apnoea: To treat or not to treat?. Eur. Respir. Rev..

[13] Dodds S., Williams L.J., Roguski A., Vennelle M., Douglas N.J., Kotoulas S.-C., Riha R.L. (2020). Mortality and morbidity in obstructive sleep apnoea-hypopnoea syndrome: Results from a 30-year prospective cohort study. ERJ Open Res..

[14] Norrie K. (1991). The Age of Legal Capacity (Sc) Act 1991. J. Law Soc. Scotl..

[15] Hill E.A., Fairley D.M., McConnell E., Morrison I., Celmiņa M., Kotoulas S.-C., Riha R.L. (2020). Utility of the pictorial Epworth sleepiness scale in the adult down syndrome population. Sleep Med..

[16] Ghiassi R., Murphy K., Cummin A.R., Partridge M.R. (2010). Developing a pictorial Epworth Sleepiness Scale. Thorax.

[17] Iber C., Ancoli-Israel S., Chesson A.L., Quan S.F. (2007). The AASM Manual for the Scoring of Sleep and Associated Events: Rules, Terminology and Technical Specifications.

[18] Rosen I.M., Kirsch D.B., Carden K.A., Malhotra R.K., Ramar K., Aurora R.N., Kristo D.A., Martin J.L., Olson E.J., Rosen C.L. (2018). American Academy of Sleep Medicine Board of Directors. Clinical Use of a Home Sleep Apnea Test: An Updated American Academy of Sleep Medicine Position Statement. J. Clin. Sleep Med..

[19] Schmidt C., Peigneux P., Ecajochen C., Collette F. (2012). Adapting Test Timing to the Sleep-Wake Schedule: Effects on Diurnal Neurobehavioral Performance Changes in Young Evening and Older Morning Chronotypes. Chronobiol. Int..

[20] Edgin J.O., Mason G.M., Allman M.J., Capone G.T., DeLeon I.G., Maslen C., Reeves R.H., Sherman S.L., Nadel L. (2010). Development and validation of the Arizona Cognitive Test Battery for Down syndrome. J. Neurodev. Disord..

[21] Brooks R.R., de Charro F. (2003). The Measurement and Valuation of Health Status Using EQ-5D: A European Perspective.

[22] Hays R.D., Cathy D. (1993). Sherbourne and Rebecca Mazel. The RAND 36-Item Health Survey 1.0.

[23] Bédard M., Molloy D.W., Squire L., Dubois S., Lever J.A., O’Donnell M. (2001). The Zarit Burden Interview: A new short version and screening version. Gerontologist.

[24] Mohr C., Tonge B.J., Einfeld S.L. (2005). The development of a new measure for the assessment of psychopathology in adults with intellectual disability. J. Intellect. Disabil. Res..

[25] Goldberg D.P. (1972). The Detection of Psychiatric Illness by Questionnaire.

[26] McFadyen T.A., Espie C.A., McArdle N., Douglas N.J., Engleman H.M. (2001). Controlled, prospective trial of psychosocial function before and after continuous positive airway pressure therapy. Eur. Respir. J..

[27] Weaver T.E., Grunstein R.R. (2008). Adherence to Continuous Positive Airway Pressure Therapy: The Challenge to Effective Treatment. Proc. Am. Thorac. Soc..

[28] Engleman H.M., Kingshott R.N., Wraith P.K., Mackay T.W., Deary I.J., Douglas N.J. (1999). Randomized Placebo-controlled Crossover Trial of Continuous Positive Airway Pressure for Mild Sleep Apnea/Hypopnea Syndrome. Am. J. Respir. Crit. Care Med..

[29] Marrone O., Resta O., Salvaggio A., Giliberti T., Stefano A., Insalaco G. (2004). Preference for fixed or automatic CPAP in patients with obstructive sleep apnea syndrome. Sleep Med..

[30] Siccoli M.M., Pepperell J.C., Kohler M., Craig S.E., Davies R.J., Stradling J.R. (2008). Effects of Continuous Positive Airway Pressure on Quality of Life in Patients With Moderate to Severe Obstructive Sleep Apnea: Data From a Randomized Controlled Trial. Sleep.

[31] Trois M.S., Capone G.T., Lutz J.A., Melendres M.C., Schwartz A.R., A Collop N., Marcus C.L. (2009). Obstructive Sleep Apnea in Adults with Down Syndrome. J. Clin. Sleep Med..

[32] Marcus C.L., Radcliffe J., Konstantinopoulou S., Beck S.E., Cornaglia M.A., Traylor J., DiFeo N., Karamessinis L.R., Gallagher P.R., Meltzer L.J. (2012). Effects of Positive Airway Pressure Therapy on Neurobehavioral Outcomes in Children with Obstructive Sleep Apnea. Am. J. Respir. Crit. Care Med..

[33] McMillan A., Bratton D.J., Faria R., Laskawiec-Szkonter M., Griffin S., Davies R.J., Nunn A.J., Stradling J.R., Riha R.L., Morrell M.J. (2014). Continuous positive airway pressure in older people with obstructive sleep apnoea syndrome (PREDICT): A 12-month, multicentre, randomised trial. Lancet Respir. Med..

[34] Breslin J.H. (2011). Sleep Disturbance, Cognition, and Behavior in Down Syndrome. Ph.D. Thesis.

[35] Meng L., Benedetti A., Lafontaine A.L., Mery V., Robinson A.R., Kimoff J., Gros P., Kaminska M. (2020). Obstructive sleep apnea, CPAP therapy and Parkinson’s disease motor function: A longitudinal study. Parkinsonism Relat. Disord..

[36] Dong R., Dong Z., Liu H., Shi F., Du J. (2018). Prevalence, Risk Factors, Outcomes, and Treatment of Obstructive Sleep Apnea in Patients with Cerebrovascular Disease: A Systematic Review. J. Stroke Cerebrovasc. Dis..

[37] McDonald K.E., Schwartz N.M., Gibbons C.M., Olick R.S. (2015). “You can’t be cold and scientific”: Community views on ethical issues in intellectual disability research. J. Empir. Res. Hum. Res. Ethics..

[38] McDonald K.E., Kidney C.A., Patka M. (2013). ‘You need to let your voice be heard’: Research participants’ views on research. J. Intellect. Disabil. Res..

[39] Massie C.A., Hart R.W., Peralez K., Richards G.N. (1999). Effects of humidification on nasal symptoms and compliance in sleep apnea patients using continuous positive airway pressure. Chest.

[40] Worsnop C., Miseski S., Rochford P.D. (2010). Routine use of humidification with nasal continuous positive airway pressure. Intern. Med. J..

[41] Vennelle M., White S., Riha R.L., Mackay T.W., Engleman H.M., Douglas N.J. (2010). Randomized Controlled Trial of Variable-Pressure Versus Fixed-Pressure Continuous Positive Airway Pressure (CPAP) Treatment for Patients with Obstructive Sleep Apnea/Hypopnea Syndrome (OSAHS). Sleep.

[42] Hoy C.J., Vennelle M., Kingshott R.N., Engleman H.M., Douglas N.J. (1999). Can Intensive Support Improve Continuous Positive Airway Pressure Use in Patients with the Sleep Apnea/Hypopnea Syndrome?. Am. J. Respir. Crit. Care Med..

[43] Stepnowsky C., Palau J.J., Gifford A.L., Ancoli-Israel S. (2007). A Self-Management Approach to Improving Continuous Positive Airway Pressure Adherence and Outcomes. Behav. Sleep Med..

[44] Bennett F., Eisenman P., French R., Henderson H., Shultz B. (1989). The Effect of a Token Economy on the Exercise Behavior of Individuals with Down Syndrome. Adapt. Phys. Act. Q..

